# Implementation of a standardized electronic medical record-integrated alternating hyperosmolar therapy protocol in neurocritical care: a single-center retrospective feasibility study

**DOI:** 10.3389/fneur.2026.1901580

**Published:** 2026-09-08

**Authors:** Myriam Semaan, Dania Ghaziri, Wassim Nasreddine, Tarek El Halabi

**Affiliations:** 1 Department of Neurology, American University of Beirut Medical Center, Beirut, Lebanon; 2 Department of Pharmacy, American University of Beirut Medical Center, Beirut, Lebanon

**Keywords:** cerebral edema, clinical protocol, feasibility, hyperosmolar therapy, hypertonic saline, intracranial hypertension, mannitol, neurocritical care

## Abstract

**Background:**

Hyperosmolar therapy is a cornerstone of the medical management of cerebral edema and suspected intracranial hypertension. Despite widespread use of hypertonic saline and mannitol, substantial variability exists in agent selection, dosing, laboratory monitoring, treatment duration, and discontinuation practices. Standardized institutional protocols may improve consistency of care; however, reports describing their implementation in routine neurocritical care remain limited.

**Objective:**

To evaluate implementation of a standardized electronic medical record (EMR)-integrated alternating hyperosmolar therapy protocol in a neurocritical care unit and to describe protocol exposure, reasons for protocol discontinuation, and preliminary safety observations.

**Methods:**

We conducted a retrospective single-center observational study of consecutive adult patients managed using a standardized alternating hyperosmolar therapy protocol between January 2024 and January 2026. The protocol consisted of alternating 3% hypertonic saline and 20% mannitol every 12 h according to a predefined four-day de-escalating schedule integrated into the institutional EMR. Implementation was assessed descriptively using treatment exposure, protocol completion, and primary reasons for protocol discontinuation; no prespecified feasibility threshold was used. Secondary outcomes included adverse events and in-hospital mortality.

**Results:**

Seventeen patients were included. Six (35.3%) completed the four-day protocol, and 16 (94.1%) received at least two days of therapy. Protocol discontinuation most commonly reflected progression of the underlying neurological disease or changes in overall goals of care rather than treatment-related toxicity. Hypernatremia occurred in three patients (17.6%) and resulted in protocol discontinuation in one. Elevated serum osmolality occurred in one patient (5.9%). No new-onset protocol-related acute kidney injury, pulmonary edema, or decompensated heart failure occurred during protocol administration. Rebound cerebral edema could not be systematically assessed because invasive intracranial pressure monitoring and protocolized follow-up neuroimaging were not routinely performed. Overall in-hospital mortality was 52.9%.

**Conclusion:**

Implementation of a standardized EMR-integrated alternating hyperosmolar therapy protocol was achievable in this retrospective single-center cohort. Most protocol discontinuations reflected progression of the underlying neurological disease or changes in overall goals of care rather than treatment-related toxicity. Although adverse events were infrequent, the retrospective design, absence of a comparator group, and small sample size preclude definitive conclusions regarding safety or efficacy. These findings support the feasibility of implementation and future prospective multicenter evaluation.

## Introduction

Elevated intracranial pressure resulting from cerebral edema is a major cause of secondary neurological injury and contributes substantially to morbidity and mortality after acute brain injury. It may complicate intracerebral hemorrhage, traumatic brain injury, aneurysmal subarachnoid hemorrhage, malignant ischemic stroke, and intracranial neoplasms. Early recognition and timely treatment of suspected intracranial hypertension are therefore fundamental objectives of neurocritical care (1, 2).

Hyperosmolar therapy is a cornerstone of the medical management of cerebral edema and suspected intracranial hypertension. Hypertonic saline and mannitol are the two most frequently administered hyperosmolar agents and are supported by contemporary neurocritical care guidance for appropriately selected patients (1, 2). Although both agents reduce intracranial pressure, available evidence has not established consistent superiority of one agent or a clear improvement in long-term functional outcomes. Treatment decisions therefore remain individualized according to the neurological disorder, patient-specific factors, institutional practice, and clinician judgment (1–4).

Considerable variability persists regarding agent selection, dosing, laboratory monitoring, treatment duration, and discontinuation criteria. Even within individual institutions, patients with similar presentations may receive different regimens depending on the treating physician. Such variability may complicate multidisciplinary communication, hinder standardized monitoring, and reduce consistency of care. Contemporary consensus recommendations acknowledge this heterogeneity and preserve individualized management because high-quality comparative evidence remains limited (1, 3).

Standardized clinical pathways have been used in critical care to reduce unwarranted practice variation, improve adherence to evidence-informed care, facilitate multidisciplinary communication, and support quality-improvement initiatives (5, 6). However, few reports have described an electronic medical record-integrated institutional protocol guiding the prescribing, monitoring, and de-escalation of hyperosmolar therapy in routine neurocritical care.

Hypertonic saline and mannitol have distinct pharmacological effects that may offer complementary practical advantages. Hypertonic saline expands intravascular volume and increases serum sodium, whereas mannitol reduces brain water through osmotic diuresis but may contribute to hypovolemia and renal dysfunction with repeated exposure (1, 7, 8). Our institution developed a standardized electronic medical record-integrated protocol incorporating alternating 3% hypertonic saline and 20% mannitol in a predefined four-day de-escalating schedule. The protocol was intended to reduce prescribing variability and standardize monitoring while preserving clinician discretion. It was not designed to establish superiority of alternating therapy over monotherapy.

The primary objective of this study was to evaluate implementation of a standardized electronic medical record (EMR)-integrated alternating hyperosmolar therapy protocol in a neurocritical care unit. Implementation was assessed descriptively by treatment exposure, protocol completion, and primary reasons for protocol discontinuation; no prespecified feasibility threshold was used. Secondary objectives were to describe preliminary safety observations associated with protocol implementation. Given the retrospective design and absence of a comparator group, the study was not intended to evaluate clinical efficacy or comparative safety.

## Materials and methods

### Study design and setting

We conducted a retrospective single-center observational study in the Neurocritical Care Unit at the American University of Beirut Medical Center, a tertiary academic referral center in Beirut, Lebanon. Consecutive adult patients managed using the standardized alternating hyperosmolar therapy protocol between January 2024 and January 2026 were included. The protocol was developed by a multidisciplinary team comprising neurointensivists, vascular neurologists, neurosurgeons, and clinical pharmacists and incorporated into the institutional electronic medical record as a standardized order set. The study is reported in accordance with the Strengthening the Reporting of Observational Studies in Epidemiology statement (9).

### Patient selection

Eligible patients were adults aged 18 years or older with an acute neurological disorder associated with clinical and/or radiographic evidence suggestive of intracranial hypertension requiring hyperosmolar therapy. Underlying diagnoses included intracranial hemorrhage, traumatic brain injury, aneurysmal subarachnoid hemorrhage, malignant ischemic stroke, and tumor-associated cerebral edema.

Clinical evidence suggestive of intracranial hypertension included neurological deterioration, worsening level of consciousness, new pupillary abnormalities, progressive focal neurological deficits, or other signs concerning for impending cerebral herniation. Radiographic evidence included cerebral edema associated with mass effect, midline shift, ventricular compression, sulcal effacement, or basal cistern effacement on computed tomography or magnetic resonance imaging.

Because invasive intracranial pressure monitoring was not routinely performed in this population, initiation of hyperosmolar therapy was based on the treating neurointensivist’s clinical assessment together with neuroimaging findings, consistent with contemporary recommendations for individualized management of cerebral edema (1).

### Bias and missing data

To reduce selection bias, all consecutive adult patients who received hyperosmolar therapy for cerebral edema or suspected intracranial hypertension during the study period were identified through the institutional EMR and included. The standardized order set was used for all otherwise eligible patients during this period, and no eligible patient received an alternative hyperosmolar regimen outside the protocol. No statistical imputation was performed. Core demographic, protocol-exposure, safety, and mortality outcomes were available for all 17 included patients; discharge Glasgow Coma Scale data were available only for survivors.

### Standardized alternating hyperosmolar therapy protocol

The institutional protocol consisted of alternating administrations of 3% hypertonic saline and 20% mannitol every 12 h according to a predefined four-day de-escalating schedule incorporated into the institutional electronic medical record. The alternating strategy, four-day duration, and stepwise dose reductions were developed pragmatically on the basis of institutional clinical experience and multidisciplinary consensus among neurointensivists, vascular neurologists, neurosurgeons, and clinical pharmacists; they were not derived from comparative evidence establishing an optimal treatment duration. Treatment was initiated with 200 mL of 3% hypertonic saline alternating with 40 g of 20% mannitol during the first 24 h. Doses were reduced to 150 mL and 30 g on Day 2, 100 mL and 20 g on Day 3, and 50 mL and 10 g on Day 4.

Patients who received intraoperative mannitol before admission to the Neurocritical Care Unit initiated the protocol at the Day 2 dosing level. Fixed mannitol doses were adopted to simplify prescribing and administration within a reproducible EMR-integrated de-escalation schedule; this pragmatic approach was not intended to imply superiority over weight-based dosing. The protocol standardized prescribing and monitoring while preserving clinician discretion. Treating physicians retained authority to modify, discontinue, or escalate therapy according to the patient’s evolving clinical condition.

### Clinical monitoring

Patients underwent serial neurological examinations and routine laboratory monitoring. During protocol administration, serum sodium was measured twice daily, while measured serum osmolality and serum creatinine were assessed once daily. Blood urea nitrogen, serum glucose, and hemodynamic parameters were also monitored as part of routine neurocritical care. The institutional therapeutic targets were a serum sodium concentration of 140–150 mmol/L and measured serum osmolality of 310–320 mOsm/kg. Hypernatremia was defined as serum sodium >150 mmol/L. Treatment modification or discontinuation was determined by the treating physician according to laboratory findings and the overall clinical condition.

### Outcome measures

Implementation was assessed descriptively by treatment exposure, protocol completion, and the primary reason for protocol discontinuation. No prespecified feasibility threshold was used. For patients who did not complete the four-day protocol, discontinuation categories represented the primary reason and were mutually exclusive. Secondary outcomes included hypernatremia, elevated serum osmolality, new-onset acute kidney injury after protocol initiation, pulmonary edema, decompensated heart failure, clinically and radiographically suspected rebound cerebral edema, in-hospital mortality, and neurological status.

Elevated serum osmolality was defined as measured serum osmolality exceeding the institutional target. New-onset acute kidney injury was assessed according to the serum creatinine component of the Kidney Disease: Improving Global Outcomes (KDIGO) criteria and was defined as an increase in serum creatinine of ≥0.3 mg/dL within 48 h or to ≥1.5 times the baseline value within seven days (10). Urine-output criteria were not included in this analysis. A patient meeting AKI criteria before protocol initiation was not classified as having new-onset AKI during protocol treatment. Rebound cerebral edema was defined as neurological deterioration associated with radiographic worsening of cerebral edema after reduction or discontinuation of hyperosmolar therapy.

Neurological status was assessed using the Glasgow Coma Scale at protocol initiation and hospital discharge. Because discharge Glasgow Coma Scale findings were available only among survivors, these observations were analyzed descriptively and were not used to infer treatment efficacy.

### Statistical analysis

Continuous variables are reported as mean ± standard deviation or median (range), as appropriate. Categorical variables are reported as frequencies and percentages. Exact binomial 95% confidence intervals were calculated using the Clopper-Pearson method. Analyses were descriptive, and no hypothesis testing was performed. No formal sample-size calculation was performed; the study included all eligible patients treated with the protocol during the predefined study period.

### Ethics statement

The study was approved by the Institutional Review Board of the American University of Beirut Medical Center (Protocol No. BIO-2025-0461). Written informed consent was waived by the Institutional Review Board because of the retrospective study design. In accordance with Institutional Review Board requirements, oral informed consent was obtained from eligible living participants whenever feasible. The study was conducted in accordance with the Declaration of Helsinki (11).

## Results

### Patient characteristics

Between January 2024 and January 2026, 17 consecutive adult patients managed using the protocol were included. The mean age was 62.2 ± 17.6 years (median, 64 years; range, 24–90 years), and nine patients (52.9%) were female. Intracranial hemorrhage was the most common underlying diagnosis, accounting for 11 patients (64.7%), including aneurysmal subarachnoid hemorrhage (*n* = 3) and other intracranial hemorrhages (*n* = 8). Additional etiologies included tumor-associated cerebral edema (*n* = 3, 17.6%), traumatic brain injury (*n* = 2, 11.8%), and malignant cerebral infarction (*n* = 1, 5.9%) (Table 1).

**Table 1:** Baseline characteristics, protocol exposure, reasons for protocol discontinuation, and safety outcomes.

Characteristic	Value (*N* = 17)
Baseline characteristics	
Age, mean ± SD, years	62.2 ± 17.6
Median age (range), years	64 (24–90)
Female sex, *n* (%)	9 (52.9)
Underlying neurological diagnosis, *n* (%)	
Intracranial hemorrhage*	11 (64.7)
Aneurysmal subarachnoid hemorrhage	3 (17.6)
Other intracranial hemorrhage	8 (47.1)
Tumor-associated cerebral edema	3 (17.6)
Traumatic brain injury	2 (11.8)
Malignant cerebral infarction	1 (5.9)
Protocol exposure, *n* (%)	
Completed 4 days	6 (35.3)
Completed 3 days	4 (23.5)
Completed 2 days	6 (35.3)
Completed 1 day	1 (5.9)
Received ≥2 days	16 (94.1)
Started at day 2 after intraoperative mannitol	3 (17.6)
Reasons for discontinuation, *n* (%)	
Progression of neurological disease/changes in goals of care	4 (23.5)
Rapid neurological deterioration or death	2 (11.8)
Treating physician-directed protocol modification	3 (17.6)
Hypernatremia	1 (5.9)
Elevated serum osmolality	1 (5.9)
Safety outcomes, *n* (%)	
Hypernatremia (>150 mmol/L)	3 (17.6)
Elevated serum osmolality**	1 (5.9)
New-onset AKI according to serum creatinine-based KDIGO criteria	0
Pulmonary edema	0
Decompensated heart failure	0
Rebound cerebral edema***	Not systematically ascertained
In-hospital mortality	9 (52.9)

*Includes aneurysmal subarachnoid hemorrhage and other intracranial hemorrhages. SD, standard deviation.

**Elevated serum osmolality was defined as serum osmolality exceeding the predefined protocol target range (310–320 mOsm/kg).

***Rebound cerebral edema was not systematically ascertained because invasive intracranial pressure monitoring and protocolized follow-up neuroimaging were not routinely performed. Discontinuation categories represent the mutually exclusive primary reason for discontinuation among the 11 patients who did not complete the four-day protocol. AKI was assessed using the serum creatinine component of the KDIGO criteria; serum creatinine was measured once daily.

### Protocol exposure and implementation

Six patients (35.3%; 95% confidence interval, 14.2–61.7%) completed the full four-day protocol. Four patients (23.5%) completed three days, six (35.3%) completed two days, and one (5.9%) received one day. Overall, 16 of 17 patients (94.1%; 95% confidence interval, 71.3–99.9%) received at least two days of protocol therapy, and 10 patients (58.8%; 95% confidence interval, 32.9–81.6%) completed three or more days. Three patients initiated the protocol at the Day 2 dosing level after intraoperative mannitol administration.

The mutually exclusive primary reasons for discontinuation were progression of the underlying neurological injury with limitation of ongoing treatment (*n* = 4), rapid neurological deterioration or death (*n* = 2), treating physician-directed protocol modification (*n* = 3), hypernatremia (*n* = 1), and elevated serum osmolality (*n* = 1) (Table 1). Treating physician-directed modification referred to clinician-initiated deviation from or discontinuation of the scheduled regimen in response to the evolving clinical condition, without a documented prespecified protocol toxicity. The specific modifications and their rationale could not be subclassified reliably from the retrospective documentation.

### Safety outcomes

Hypernatremia occurred in three patients (17.6%; 95% confidence interval, 3.8–43.4%) and resulted in protocol discontinuation in one. The other two continued treatment with close laboratory monitoring after correction of serum sodium concentrations. Measured serum osmolality exceeded the institutional target in one patient (5.9%; 95% confidence interval, 0.1–28.7%), resulting in discontinuation.

No patient met the serum creatinine-based KDIGO criteria for new-onset acute kidney injury during protocol administration. One patient had acute kidney injury before protocol initiation and was not classified as having new-onset AKI. No pulmonary edema or decompensated heart failure was documented during protocol administration. Based on retrospective review of the available clinical documentation, no death was considered directly attributable to the protocol; however, formal independent causality adjudication was not performed. Clinically or radiographically suspected rebound cerebral edema was not systematically ascertained because invasive intracranial pressure monitoring and protocolized follow-up neuroimaging were not routinely performed.

### Neurological outcomes

Nine patients died during hospitalization; therefore, Glasgow Coma Scale findings are presented only for the eight survivors (Table 2). Baseline GCS may have been influenced by sedation, mechanical ventilation, and acute critical illness, whereas discharge assessments were generally obtained after sedation had been reduced or discontinued. One patient remained in the mild category. Among three patients with moderate baseline impairment, two improved to the mild category and one remained moderate. Among four patients with severe baseline impairment, three improved to the moderate or mild category and one remained severe. Together with survivorship bias, these factors preclude attributing the observed changes to the protocol; these observations are descriptive and should not be interpreted as evidence of treatment efficacy.

**Table 2:** Glasgow Coma Scale (GCS) category among survivors.

Baseline GCS category	Discharge mild (13–15)	Discharge moderate (9–12)	Discharge severe (3–8)
Mild (13–15)	1	0	0
Moderate (9–12)	2	1	0
Severe (3–8)	1	2	1

GCS, Glasgow Coma Scale. Neurological outcomes are presented only for survivors (*n* = 8). Patients who died during hospitalization (*n* = 9) were excluded because discharge GCS was unavailable. These findings are descriptive and should not be interpreted as evidence of treatment efficacy.

### In-hospital mortality

Overall, in-hospital mortality was 52.9% (9/17; 95% confidence interval, 27.8–77.0%). Most deaths occurred in patients with severe primary neurological injury complicated by progressive cerebral edema and brain herniation. Based on retrospective review of the available clinical documentation, no death was considered directly attributable to the protocol; however, formal independent causality adjudication was not performed, and no causal relationship between the protocol and mortality can be inferred from this uncontrolled observational study.

## Discussion

This retrospective single-center study evaluated the feasibility of implementing a standardized electronic medical record-integrated alternating hyperosmolar therapy protocol. Most patients received at least two days of protocol-directed therapy. Discontinuation was more frequently related to progression of the underlying neurological disorder or changes in goals of care than to treatment-related toxicity. Adverse events were infrequent in this cohort, although the small sample size precludes definitive safety conclusions.

Hyperosmolar therapy remains central to the medical management of cerebral edema and suspected intracranial hypertension. The Neurocritical Care Society guideline recognizes both hypertonic saline and mannitol as treatment options but does not identify a single optimal regimen because comparative evidence is limited (1). The Seattle International Severe Traumatic Brain Injury Consensus Conference similarly places osmotherapy within a tiered approach that requires escalation or de-escalation according to the patient’s response rather than adherence to a rigid calendar-based pathway (3). Our protocol was therefore implemented as a clinical framework, not as a substitute for individualized decision-making.

The alternating strategy and four-day de-escalating schedule were selected pragmatically on the basis of institutional experience and multidisciplinary consensus. Hypertonic saline and mannitol have different volume and renal effects: hypertonic saline expands intravascular volume and increases serum sodium, whereas mannitol causes osmotic diuresis and may contribute to hypovolemia or renal dysfunction (1, 7, 8). The schedule was intended as a structured de-escalation framework and did not imply that cerebral edema resolves predictably over four days. Fixed mannitol doses simplified implementation within the EMR order set but were not intended to replace weight-based dosing in other settings. Treating clinicians retained discretion to stop, modify, or escalate treatment.

The present findings should not be interpreted as evidence of efficacy. In the COBI randomized clinical trial, continuous 20% hypertonic saline did not improve neurological status at six months compared with standard care in patients with moderate to severe traumatic brain injury (12). Guideline and review literature likewise has not established a consistent long-term functional benefit or clear superiority of one hyperosmolar agent over another (1, 8). The contribution of the current study is therefore implementation-focused: it describes treatment exposure, protocol completion, reasons for protocol discontinuation, and preliminary safety observations following implementation of a standardized order set.

Protocol completion alone should not be interpreted as the sole measure of feasibility. Only six patients completed all four days, but 16 received at least two days. In this severely ill population, early discontinuation often reflected deterioration, death, or limitation of treatment rather than an inability to prescribe or administer the protocol. Nevertheless, because feasibility thresholds were not prespecified, the results should be regarded as descriptive rather than proof of high feasibility.

Hypernatremia occurred in three patients, and one patient discontinued therapy for this reason. One patient discontinued because of elevated serum osmolality. No new-onset acute kidney injury was documented after protocol initiation; however, the cohort was too small to exclude clinically important uncommon harms. The absence of invasive intracranial pressure monitoring and systematic serial imaging also limits conclusions about physiological response during de-escalation.

This study has several limitations. It was retrospective, single-center, and included only 17 patients, limiting generalizability and precision. Although all eligible patients receiving hyperosmolar therapy during the study period were included and no alternative regimen was used, retrospective EMR-based ascertainment remains susceptible to documentation and measurement bias. Body weight was not available for *post hoc* analysis, and the protocol used fixed doses rather than individualized weight-based mannitol dosing; consequently, dose exposure in grams per kilogram could not be assessed, and the regimen may not be directly generalizable to centers using weight-based dosing. Baseline disease-specific severity scores, treatment intensity, higher-tier intracranial pressure interventions, withdrawal of life-sustaining treatment, and intensive care and hospital length of stay were not systematically collected. In addition, clinically or radiographically suspected rebound cerebral edema could not be systematically ascertained because invasive intracranial pressure monitoring and protocolized follow-up neuroimaging were not routinely performed. Finally, survivor-only Glasgow Coma Scale outcomes are affected by survivorship bias and cannot support inference of neurological benefit. Baseline Glasgow Coma Scale scores may also have been influenced by sedation and mechanical ventilation, whereas discharge assessments were obtained after sedation had been discontinued. Consequently, apparent improvement among survivors may partially reflect resolution of sedation rather than neurological recovery.

Prospective multicenter studies should define feasibility thresholds in advance, capture all eligible patients and off-protocol treatment, incorporate standardized severity measures, and evaluate clinician override, rescue therapies, and adverse events using prespecified definitions. Studies with invasive or noninvasive physiological monitoring are also needed to determine whether protocolized schedules adequately respond to heterogeneous edema trajectories.

## Conclusion

Implementation of a standardized electronic medical record-integrated alternating hyperosmolar therapy protocol was achievable in this small single-center cohort. Most discontinuations were related to progression of the underlying neurological disease or changes in goals of care rather than treatment-related toxicity. Because no feasibility threshold was prespecified, these descriptive findings support the feasibility of implementation but do not establish high feasibility. The retrospective design, absence of a comparator group, and limited sample size preclude conclusions regarding efficacy or definitive safety. These findings support prospective multicenter studies evaluating the implementation, safety, and clinical effectiveness of standardized hyperosmolar therapy protocols.

## Data Availability

The original contributions presented in the study are included in the article/supplementary material, further inquiries can be directed to the corresponding author/s.

## References

[1] Cook AM Morgan Jones G Hawryluk GWJ Mailloux P McLaughlin D Papangelou A . Guidelines for the acute treatment of cerebral edema in neurocritical care patients. Neurocrit Care. (2020) 32:647–66. doi: 10.1007/s12028-020-00959-7, 32227294 PMC7272487

[2] Carney N Totten AM O'Reilly C Ullman JS Hawryluk GWJ Bell MJ . Guidelines for the management of severe traumatic brain injury, fourth edition. Neurosurgery. (2017) 80:6–15. doi: 10.1227/NEU.0000000000001432, 27654000

[3] Hawryluk GWJ Aguilera S Buki A Bulger E Citerio G Cooper DJ . A management algorithm for patients with intracranial pressure monitoring: the Seattle international severe traumatic brain injury consensus conference (SIBICC). Intensive Care Med. (2019) 45:1783–94. doi: 10.1007/s00134-019-05805-9, 31659383 PMC6863785

[4] Chesnut RM Temkin N Carney N Dikmen S Rondina C Videtta W . A trial of intracranial-pressure monitoring in traumatic brain injury. N Engl J Med. (2012) 367:2471–81. doi: 10.1056/NEJMoa1207363, 23234472 PMC3565432

[5] Pronovost PJ Berenholtz SM Goeschel CA Needham DM Sexton JB Thompson DA . Creating high reliability in health care organizations. Health Serv Res. (2006) 41:1599–617. doi: 10.1111/j.1475-6773.2006.00567.x, 16898981 PMC1955341

[6] National Academy of Medicine. Crossing the Quality Chasm: A New Health System for the 21st Century. Washington, DC: National Academies Press (2001). 25057539

[7] Koenig MA. Management of raised intracranial pressure and cerebral edema. Nat Rev Neurol. (2018) 14:323–34. doi: 10.1038/s41582-018-0008-5

[8] Boone MD Oren-Grinberg A Robinson TM Chen CC Kasper EM. Mannitol or hypertonic saline in the setting of traumatic brain injury: what have we learned? Surg Neurol Int. (2015) 6:177. doi: 10.4103/2152-7806.170248, 26673517 PMC4665128

[9] von Elm E Altman DG Egger M Pocock SJ Gøtzsche PC Vandenbroucke JP . The strengthening the reporting of observational studies in epidemiology statement: guidelines for reporting observational studies. PLoS Med. (2007) 4:e296. doi: 10.1371/journal.pmed.0040296 PMC2020495 17941714

[10] Kidney Disease: Improving Global Outcomes (KDIGO) Acute Kidney Injury Work Group. KDIGO clinical practice guideline for acute kidney injury. Kidney Int Suppl. (2012) 2:1–138.

[11] World Medical Association. World medical association declaration of Helsinki: ethical principles for medical research involving human subjects. JAMA. (2013) 310:2191–4. doi: 10.1001/jama.2013.281053, 24141714

[12] Roquilly A Moyer JD Huet O Lasocki S Cohen B Dahyot-Fizelier C . Effect of continuous infusion of hypertonic saline vs standard care on 6-month neurological outcomes in patients with traumatic brain injury: the COBI randomized clinical trial. JAMA. (2021) 325:2056–66. doi: 10.1001/jama.2021.5561, 34032829 PMC8150692

